# Two Cases of Methemoglobinemia Secondary to Favism in Pediatric Patients With Unknown Glucose-6-Phosphate Dehydrogenase (G6PD) Deficiency

**DOI:** 10.7759/cureus.101222

**Published:** 2026-01-10

**Authors:** Marah A Sawaftah, Juman Babi, Heba A Abuzayda, Gamal Ahmed

**Affiliations:** 1 Pediatric Medicine, Sheikh Shakhbout Medical City, Abu Dhabi, ARE; 2 Pediatric Intensive Care, Assiut University Children Hospital, Assiut, EGY

**Keywords:** acquired methemoglobinemia, favism, glucose-6-phosphate dehydrogenase (g6pd) deficiency, l-ascorbic acid, methylene blue treatment

## Abstract

Glucose-6-phosphate dehydrogenase (G6PD) deficiency predisposes to acute hemolytic crisis when exposed to oxidative stress. Methemoglobinemia occurs as a result of the oxidation of hemoglobin. This eventually affects the oxygen-carrying capacity of the red blood cells, resulting in hypoxemia. There is a reported association between the two conditions.

A 12-year-old boy with autism, with no history of hematologic disease, presented with acute hemolysis and respiratory distress. His arterial blood gas showed a methemoglobin level of 6.1%; he received methylene blue in the emergency department. The patient’s condition deteriorated with a progressive drop in hemoglobin level. Laboratory investigations confirmed G6PD deficiency later on; it was then revealed that he had eaten fava beans one day before the symptoms. He required a blood transfusion, with an average stay of four days in the pediatric ICU, and eventually made a full recovery.

A 14-year-old boy, with short stature on growth hormone therapy and no history of hematologic disease, presented with acute hemolysis and respiratory distress one day after consuming Fava beans. His arterial blood gas showed a methemoglobinemia level of 7%; he was transfused and then transferred to another facility, where he received 4,000 mg of ascorbic acid in divided doses and multiple blood transfusions. Laboratory investigations confirmed G6PD deficiency. The patient required a blood transfusion, with an average stay of three days in the pediatric ICU, and eventually made a full recovery.

The co-occurrence of methemoglobinemia with G6PD deficiency-related Favism is rare, with few reported cases in the literature. This association can be explained by the defective nicotinamide adenine dinucleotide phosphate-dependent pathway in G6PD deficiency. This pathway helps maintain iron in the ferrous form in the blood. The methemoglobinemia secondary to hemolysis in G6PD-deficient patients poses therapeutic challenges due to the lack of equally effective therapeutic options and the relatively long time to establish G6PD status, which may also result in false negatives in acute hemolysis. Methylene blue, the primary treatment of methemoglobinemia, is contraindicated. Ascorbic acid is considered to be a safer option and effective as an additive to blood transfusion in such cases.

## Introduction

Glucose-6-phosphate dehydrogenase (G6PD) deficiency, the most common enzyme defect in humans, is an X-linked disease caused by a mutation in the gene that encodes the G6PD enzyme [1]. This deficiency results in acute hemolytic anemia after exposure to oxidative substances such as fava beans and certain medications, including methylene blue. It is more common in African, Asian, and Mediterranean ethnicities [2]. G6PD enzyme functions to prevent the destructive effect of oxidative substances by aiding the formation of reduced glutathione through providing nicotinamide adenine dinucleotide phosphate (NADPH) as the reducing substrate. Reduced glutathione acts as a scavenger of oxidative substances. Considering its high prevalence, G6PD deficiency is now part of many newborn screening programs aiming for early detection. Nevertheless, it was initiated in 2009, and some older patients were never tested [3]. 

Methemoglobinemia is a potentially fatal condition caused by the oxidation of iron in the hemoglobin from the ferrous (Fe^2+^) to the ferric (Fe^3+^) state, affecting the hemoglobin's carrying capacity of oxygen and causing hypoxemia [4]. Methemoglobinemia can be congenital, but it is mostly acquired [5]. An association between the two conditions has been reported in a few cases. We will report two cases of methemoglobinemia secondary to fava bean ingestion in undiagnosed G6PD-deficient pediatric patients. We will compare the intervention received and the clinical course of both cases.

## Case presentation

Case 1 summary

A 12-year-old male, known only to have severe non-verbal autism, was brought by his mother to the emergency department with respiratory distress for one day. Mother initially reported that the symptoms started one day after suspected ingestion of an unknown plant at the park. Since then, the patient reported having increased work of breathing, jaundice with dark urine, and restlessness.

On examination, the patient was anxious and jaundiced with icteric sclera. He was afebrile, had difficulty breathing, a respiratory rate in the late 20s, and no added sounds on chest auscultation. His oxygen saturation ranged from 84% to 87% on room air. There was no improvement in oxygen saturation in the pulse oximeter despite receiving 15 L of oxygen via a non-breathable mask. The remainder of the physical examination was unremarkable. The initial venous blood gas was showing compensated respiratory acidosis with methemoglobin levels of 6.1% (high). Chest X-rays were unremarkable. The remaining laboratory workups were showing high bilirubin (mainly indirect), liver enzymes were within normal limits, hepatitis panel was negative, hemoglobin was 9.4 g/L, and other parameters of hemolysis were still pending. The patient then received 1 mg/kg methylene blue, as his provisional diagnosis was methemoglobinemia, and was admitted to the pediatric ICU for further support and stabilization.

In the pediatric ICU, after 1 hour of receiving methylene blue, laboratory results showed G6PD deficiency, and a subsequent arterial blood gas showed methemoglobin of 7.5%, PaO_2_ of 242, and O_2_ saturation of 101%, while the pulse oximeter showed O_2_ saturation of 80%-86%, reflecting an oxygen saturation gap. Patient vitals did not improve despite giving oxygen support of 10 L via a non-breathing mask. Later, laboratory workup was repeated, and hemoglobin kept dropping from 9.4 g/L to 7.5 g/L, then 5.7 g/L. Further investigations showed low haptoglobin associated with hemolysis. Urine started becoming bluish, so the patient got blood transfusions, and his vitals started improving with minimal support of oxygen via nasal cannula. Urine color was observed to clear out from a bluish-greenish color.

Hemoglobin after initial transfusions improved from 5.7 g/L to 7 g/L, but the next day it dropped again to 6.4 g/L; thus, another blood transfusion was given, and his oxygen support was weaned off. In total, the patient received five packed RBC transfusions; his last hemoglobin level measured was 10.4 g/L, and he was discharged home after five days of hospitalization on oral folic acid. Later on, it was revealed by the housemaid that the patient was served fava beans at school lunch the same day of symptom onset, and that was the first time he had consumed them.

Case 2 summary

A 14-year-old male, known to have short stature on growth hormone therapy, presented to the emergency department with jaundice and cola-colored urine associated with mid-abdominal pain. This presentation occurred one day after consuming fava beans for the first time. In the emergency department, the patient was found to be in respiratory distress. He was desaturating despite receiving high-flow oxygen support. Blood gas was performed in the first hospital, which showed a methemoglobin level of 10% (high), and other blood gas parameters were not available. Investigations showed a hemoglobin level of 85 g/L, and the patient then received a 250 mL blood transfusion. He was started on ceftriaxone due to fever and then transferred to another facility upon family request.

On arrival at the new facility's emergency room, the patient was still maintaining oxygen saturation of 80% despite being on oxygen support. Repeated arterial blood gas showed a methemoglobin level of 7%, a PaO_2_ of 248 mmHg, and an O_2 _saturation of 101%, which reflects an oxygen saturation gap. Initial workup showed hemoglobin of 7.7 g/L, elevated direct bilirubin, high lactate dehydrogenase, and low haptoglobin. G6PD enzyme level came low, suggesting a diagnosis of G6PD deficiency/acute hemolysis and methemoglobinemia. The patient was directly admitted to the pediatric ICU. The patient received a total of 4,000 mg of ascorbic acid, divided into four doses (1,000 mg every 6 hours).

There was an improvement in oxygen saturation immediately after receiving the first dose of ascorbic acid, and the patient was off oxygen support after four hours. Repeated blood gas one hour after starting ascorbic acid showed a methemoglobin level of 4.5%. Methemoglobin was gradually monitored afterward until its level became 1.8% on the third day of admission. Throughout the pediatric ICU stay, the patient was demonstrating signs of active hemolysis, dark colored urine, and dropping hemoglobin, which required multiple transfusions. The patient received a total of five transfusions of packed RBC. On the third day of admission, the patient’s urine started to clear, and the latest hemoglobin became 8.6 g/L. The patient was then discharged safely without complications. Table 1 demonstrates a comparison between the two cases regarding laboratory findings, clinical course, and response to initial management.

**Table 1:** The table demonstrates the difference in clinical presentation between the two cases. ALT: Alanine transaminase; AST: Aspartate aminotransferase; G6PD: Glucose-6-phosphate dehydrogenase; Hb: Hemoglobin; MetHb: Methemoglobin; NA: Not applicable; PRBC: Packed red blood cell. (1) means first reading after initiating the treatment, and (2) means second reading after initiating the treatment.

Parameters	Reference value	Case 1	Case 2
Age	NA	12 years old	14 years old
Gender	NA	Male	Male
Previous diagnosis of G6PD (G6PD status)	NA	Unknown (newly diagnosed)	Unknown (newly diagnosed)
Hb level (g/L) - Nadir	11.6-16.6	5.7	6.7
Hb level (g/L) - on discharge	11.6-16.6	10.8	8.6
Retics count (10^9^/L)	50-100	142	127.9
Platelet count (10^9^/L)	140-400	306	253
Urinary myoglobin (μg/L)	≤20	Not done	397
AST (IU/L)	≤50	98	97
ALT (IU/L)	≤50	17	31
Pre-treatment MetHb level, %	0-1.5	6.1	7
Post-treatment MetHb level, % (1)	0-1.5	7.9 (post-methylene blue)	4.5 (post-ascorbic acid)
Post-treatment MetHb level, % (2)	0-1.5	6.5	3.8
Post-treatment MetHb level, % (on discharge)	0-1.5	0.7	1.8
Total PRBC units received	NA	1280 mL	1284 mL
Therapy provided	NA	Methylene blue and blood transfusions	Ascorbic acid and blood transfusions

## Discussion

Methemoglobin is normally present in the blood in very small amounts as the hemoglobin auto-oxidizes in the presence of oxygen [4]. Methemoglobin is maintained at a physiological level, which is <1% of the total blood hemoglobin, by two pathways. The predominant pathway is by the enzyme cytochrome B reductase, which adds an electron to the hemoglobin in the ferric form (Fe^3+^), changing it to the ferrous form (Fe^2+^). The alternative minor pathway is by NADPH methemoglobin reductase. This pathway plays a negligible role but is potentiated by methylene blue therapy [6]. So, a defect in any of these pathways can increase the methemoglobin level in the blood, leading to methemoglobinemia. Acquired methemoglobinemia can be caused by exposure to certain toxins and medications such as local anesthesia, nitrites, certain antibiotics (such as dapsone and sulfonamides), and antimalarial medications [5].

Methemoglobinemia is defined as a level of more than 5%. A level of methemoglobinemia below 10% is usually not associated with any symptoms. The presentation of methemoglobinemia depends on the methemoglobin level. It can range from being asymptomatic to having non-specific symptoms such as fatigue, headache, cyanosis, progressive altered mental status, fatal cardiac arrhythmias, and death, when levels are above 70%.

Both patients demonstrated significant respiratory distress with a methemoglobin level of <10%. It is suggested that patients with acute hemolysis develop a lower threshold for methemoglobinemia-associated symptoms since they have decreased hemoglobin content in the blood [7]. 

In methemoglobinemia, oxygen saturation reading is maintained at 85% with pulse oximetry regardless of true oxygen saturation. This reading characteristically does not improve with supplemental oxygen. The methemoglobin absorbs light in distinct wavelengths, thus affecting the pulse oximetry reading. In parallel, blood gas will show falsely normal oxygen saturation level [6]. This is attributed to the fact that PaO_2_ reading is not affected by hemoglobin-oxygen binding capacity [8].

Treatment thresholds for methemoglobinemia depend on the clinical status. A level of 30% or more should be generally considered for treatment. While a level of 20% is the cut-off for treatment in symptomatic patients. In case of a significant cardiopulmonary condition, a lower level of 10% is the threshold to start therapy [9]. Methylene blue is one of the medications that can predispose to hemolytic crisis in patients with G6PD deficiency, and thus should be avoided. This poses a challenge to healthcare providers as methylene blue is considered the first-line treatment for methemoglobinemia [9]. Alternative options to treat methemoglobinemia in patients with G6PD deficiency are ascorbic acid, exchange transfusions, and hyperbaric oxygen, in addition to the management of the acute hemolytic crisis by blood transfusion [9].

It is suggested that patients with G6PD deficiency can develop methemoglobinemia secondary to favism or acute hemolytic crisis. This occurs because of a defective NADPH-dependent pathway, which helps maintain iron in the ferrous form in the blood [10]. Although the co-occurrence of both conditions is rare, there are so far 12 cases reported in the literature in both the adult and pediatric age groups [11]. All of them were found to have a fava bean ingestion history, making it the most frequent cause of this co-occurrence [12].

The patient in the first case was brought to the emergency department with the presentation of respiratory distress, which was attributed to the methemoglobinemia found in the venous blood gas. Although the patient had manifestations of acute hemolysis, jaundice, low hemoglobin, and hypoxemia, the patient was mismanaged by giving methylene blue without knowing he was G6PD deficient. This led to worsening of hemolysis, methemoglobinemia, and further increase in respiratory distress and prolonged hospital stay. On the other hand, the patient in the second case was given a transfusion immediately, as hemolytic anemia was evident in the laboratory, and then ascorbic acid was initiated. Although he required multiple transfusions for the ongoing hemolysis, in comparison to the first case, this patient had more rapid improvement in methemoglobinemia level in the blood, less demand for oxygen support, and a shorter hospital stay. Understanding this relationship between the two conditions is crucial when making decisions regarding management.

Low doses of ascorbic acid are considered to be a safer alternative to treat methemoglobinemia during G6PD deficiency acute hemolysis than methylene blue. However, high doses (>6 g/day) possess a risk of oxidative stress and acute hemolysis as well [13]. There is a reported case of a 77-year-old patient with an unrecognized G6PD deficiency, admitted with COVID-19 infection. Patients received a high dose of ascorbic acid as proposed to treat severe COVID pneumonia. This caused acute hemolysis and methemoglobinemia, which resolved after stopping ascorbic acid [13]. Among the cases reported in the literature of this co-occurrence, a few of them were given ascorbic acid for methemoglobinemia and ended in complete resolution of symptoms without further complications [12]. No guidelines have been established yet regarding managing cases with this co-occurrence, and the modality of treatment is considered the first line.

## Conclusions

Methemoglobinemia secondary to hemolysis in G6PD-deficient patients poses diagnostic challenges due to the relatively long time to establish G6PD status and the possibility of false-negative results in acute hemolysis. Therefore, clinicians should maintain a high index of suspicion for G6PD deficiency even in the absence of suggestive clinical or family history. 

Conventional treatment with methylene blue for such cases will worsen the symptoms and might lead to life-threatening complications. Ascorbic acid and exchange transfusion are preferred alternative therapeutic strategies. While transfusion of packed RBC should be considered for severe hemolysis.
